# Differences in the Clinical Characteristics and 1-Year Mortality Rates of Patients with Dermatomyositis with anti-Jo-1 and anti-MDA5 Antibodies

**DOI:** 10.1155/2023/2988422

**Published:** 2023-01-04

**Authors:** Chao-Han Liu, Chew-Teng Kor, Ming-Hui Hung, Kai-Hung Hsiao, Yen-Huang Cheng, Ya-Chih Tien

**Affiliations:** ^1^Division of Allergy, Immunology and Rheumatology, Department of Internal Medicine, Changhua Christian Hospital, Changhua, Taiwan; ^2^Big Data Center, Changhua Christian Hospital, Changhua, Taiwan; ^3^Graduate Institute of Statistics and Information Science, National Changhua University of Education, Changhua, Taiwan; ^4^Department of Emergency Medicine, Chang Bing Show Chwan Memorial Hospital, Changhua, Taiwan

## Abstract

**Objective:**

Patients with anti-Jo-1 antibodies (Abs) and anti–melanoma differentiation-associated protein 5 (MDA5) Abs are at a higher risk of interstitial lung disease (ILD) and have a mortality rate higher than that of patients with anti-Jo-1 Abs. This study investigated differences in the clinical characteristics and prognosis of patients with anti-Jo-1 Abs and anti-MDA5 Abs with dermatomyositis (DM).

**Methods:**

We retrospectively reviewed the medical records of 38 patients with DM from January 2000 to December 2021. The patients were divided into anti-Jo-1 Abs and anti-MDA5 Abs groups. The basic demographic data, clinical manifestations, and 1-year mortality rates of the groups were compared.

**Results:**

Among the 38 patients, 30 were anti-Jo-1-Abs positive and 8 patients were anti-MDA5 Aba positive. The patients with anti-MDA5 Abs presented with more apparent cutaneous symptoms and aggressive pulmonary manifestations than did those with anti-Jo-1 Abs. The mortality rate in the anti-MDA5 Abs group (1.95/person-year (PY)) was much higher than that in anti-Jo-1 Abs group (0.094/PY), and most of the mortalities occurred within the first 1–3 months of follow-up.

**Conclusion:**

Distinct cutaneous and pulmonary manifestations were observed in the anti-Jo-1 Abs and anti-MDA5 Abs groups. The mortality rate in the anti-MDA5 Abs group was significantly higher than that in the anti-Jo-1 Abs group. Early recognition is crucial to ensuring higher chances of survival for patients with anti-MDA5 Abs.

## 1. Introduction

Dermatomyositis (DM) is a systemic autoimmune disease described as an idiopathic inflammatory myopathy that affects patients' muscles, lungs, and skin to varying extents [1]. The prevalence rate of DM is approximately 1 case per 100,000 in the general population. The female to male ratio of patients with DM is approximately 2 : 1, and DM most often affects adults aged 40 to 50 years [2].

Aminoacyl-tRNA (ARS) synthetase antibodies (Abs) such as anti-Jo-1 Abs are the most common myositis-specific Abs (MSAs), accounting for approximately 25%–40% of adults with inflammatory myopathies [3, 4]. Patients with anti-Jo-1 Abs are at higher risk of interstitial lung disease (ILD) and frequently show a combination of symptoms including ILD, polyarthritis, fever, Raynaud's phenomenon, and mechanic's hands, known as antisynthetase syndrome [5].

Antimelanoma differentiation-associated protein 5 (MDA5) Abs are common in East Asia and are present in approximately 10%–20% of patients with DM. Patients with anti-MDA5 Abs are more likely to have clinical amyopathic DM (cADM) and are at a higher risk of ILD, particularly rapidly progressive ILD (RP-ILD) and ulcered skin lesions. Several studies have demonstrated that RP-ILD is refractory and fatal in patients with anti-MDA5 Abs [6, 7]. Although immunosuppressive treatment with a combination of corticosteroids, calcineurin inhibitors, and intravenous pulse cyclophosphamide (CYC) may improve the chances of survival and prognosis of patients with RP-ILD, 20%–30% of patients with RP-ILD who are treated with aggressive immunosuppressive agents still die from respiratory failure shortly after diagnosis [8–10].

Anti-Jo-1 and anti-MDA5 Abs increase the risk of ILD. However, ILD seems to be more refractory and fatal in patients with anti-MDA5 Abs, and few studies have investigated the differences between patients with these two types of MSAs. This retrospective study is aimed at clarifying the differences in the clinical characteristics and prognosis of patients with DM with anti-Jo-1 and anti-MDA5 Abs.

## 2. Materials and Methods

### 2.1. Data Source and Study Population

We retrospectively reviewed the medical records of 38 patients with DM treated at Changhua Christian Hospital from January 2000 to December 2021. DM was diagnosed by a physician according to the Bohan and Peter criteria [11, 12]. The data we collected from each patient's medical records were their gender, age at onset, signs at presentation, clinical symptoms, laboratory test results, ILD status, comorbidities, and survival status. ILD was diagnosed when a patient's high-resolution computed tomography (HRCT) findings were consistent with ILD. The HRCT images of patients with ILD indicate nonspecific interstitial pneumonia (NSIP) or NSIP with an organizing pneumonia (OP)/fibrosing OP (FOP) pattern. Mortality was defined as patient death recorded in the hospital medical records.

The Institutional Review Board of Changhua Christian Hospital reviewed and approved our study protocol (IRB No. 211203).

### 2.2. Statistical Analysis

**Figure 1:**
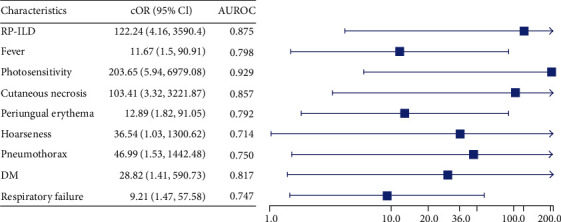
Characteristics significantly associated with anti-MDA-5-Ab-positive status.

**Figure 2:**
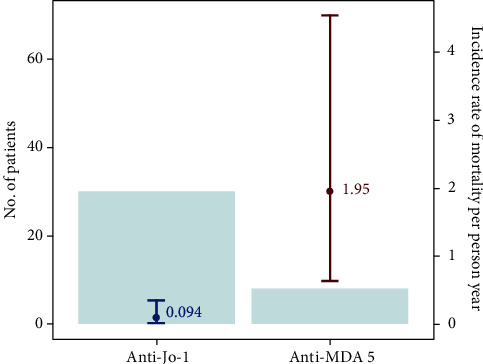
One-year mortality rate of anti-Jo-1 and anti-MDA-5 patients.

Categorical and continuous variables are expressed as numbers (proportions) and means ± standard deviations, respectively. A chi-square test was used to compare the categorical variables, and Student's *t*-test was used to compare the continuous variables. Firth's penalized likelihood approach was used to reduce the bias of the parameter estimates due to small sample size. Significant clinical characteristics were selected for further logistic regression to assess the associations of clinical characteristics with anti-MDA5 Abs. We use a forest plot to visualize the odds ratios (Figure 1). A graph of mortality rates (per person-years) during the follow-up period and the Kaplan–Meier survival curves of the patients in the anti-MDA5 Ab and anti-Jo-1 Ab groups are presented in Figure 2. All the statistical analyses were performed using SPSS software, version 22.0 (SPSS, Chicago, IL, USA), and the figures were produced using the plot2 and forest plot packages in R. A two-tailed *P* value of < 0.05 was considered statistically significant.

## 3. Results

### 3.1. Difference in the Clinical Manifestations of Patients with anti-MDA5 and anti-Jo1 Abs

A total of 38 patients with DM were included in this study. Among the 38 patients, 30 were anti-Jo1-Abs positive and the remaining 8 were anti-MDA5 Abs positive.  Table 1 presents the results of univariate analysis of the clinical manifestations of the anti-Jo-1 Abs and anti-MDA5 Abs groups. The patients with anti-MDA5 Abs presented with more apparent cutaneous symptoms and aggressive pulmonary manifestations than did those with anti-Jo-1 Abs. Figure 1 presents the characteristics, namely, RP-ILD, fever, photosensitivity, cutaneous necrosis, periungual erythema, hoarseness, pneumothorax, respiratory failure, and comorbid diabetes mellitus, significantly associated with anti-MDA5 Abs positive.

### 3.2. Mortality Rates of Patients with anti-MDA5 and anti-Jo-1 Abs

**Figure 3:**
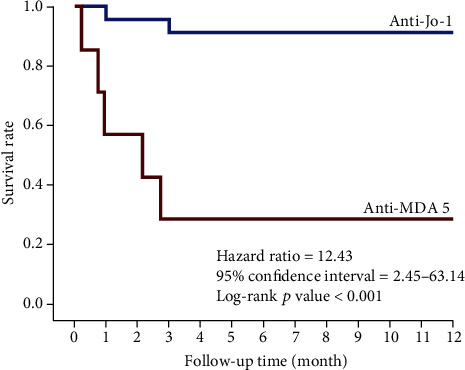
The Kaplan-Meier survival curve of anti-Jo-1 and anti-MDA-5 patients.

The mortality rate in the anti-MDA5 Abs group (1.95/person-year (PY)) was much higher than that in anti-Jo-1 Abs group (0.094/PY; Figure 2). The Kaplan–Meier survival curves revealed that the 1-year survival rate in the anti-MDA5 Abs group was significantly lower than that in the anti-Jo-1 Abs group (HR = 12.43, 95% CI = 2.45–63.14; Figure 3). The causes of death for DM patients were concomitant pulmonary infection with RP-ILD (57.1%), followed by pulmonary infection (28.6%) and ILD exacerbation (14.3%). Pathogens of pulmonary infection include PJP (*n* = 3), aspergillus (*n* = 2), and pseudomonas (*n* = 1). In another word, most of the patients expired due to respiratory failure resulting from RP-ILD and opportunistic infection after intensive immunosuppressive treatment for ILD. Most of the mortalities occurred within the first 1–3 months of follow-up.

## 4. Discussion

This is the first retrospective study to compare the clinical characteristics and prognosis of patients with DM with anti-Jo-1 and anti-MDA5 Abs. The patients with anti-MDA5 Abs had higher rates of fever, photosensitivity, cutaneous necrosis, periungual erythema, hoarseness, RP-ILD, pneumothorax, and respiratory failure than did those with anti-Jo-1 Abs. ILD and RP-ILD occur in 82%–100% and 39%–100%, respectively, of patients with anti-MDA5 Abs in East Asian populations [13]. Similarly, in our study, 87.5% of the patients in the anti-MDA5 Abs group presented with ILD, and of those, 75% presented with RP-ILD. The patients with anti-MDA5 Abs were also at a significantly higher risk of RP-ILD than were those with anti-Jo-1 Abs. In addition, the prevalence of cutaneous necrosis among the patients with anti-MDA5 Abs was higher than that among the patients with anti-Jo-1 Abs, which is consistent with a previous study's finding that anti-MDA5 Abs are associated with a unique cutaneous phenotype comprising cutaneous necrosis/ulceration, tender palmar papules, and ILD [14–16]. Cutaneous ulcers have been observed in 3%–19% of patients with anti-MDA5 Abs [17]. In a study by Cao et al. [18], patients with ulcerative Gottron papules were at a higher risk of acute or subacute interstitial pneumonia than were patients with nonulcerative Gottron papules and were more likely to test positive for anti-MDA5 Abs. Although the mechanisms underlying clinical phenotypes and the association between cutaneous ulceration and the pulmonary manifestations of anti-MDA5 Abs remain unknown [17], according to the findings of our study and other studies, patients with anti-MDA5 Abs have more apparent cutaneous lesions (such as cutaneous ulcerations), which may be positively correlated with aggressive pulmonary involvement (including RP-ILD or acute interstitial pneumonia), than those patients with anti-Jo-1 Abs. These distinct clinical manifestations may help clinicians diagnose different subtypes of DM.

In the present study, the mortality rate in the anti-MDA5 Abs group was much higher than that in anti-Jo-1 Abs group (1.95/PY vs. 0.094/PY). The 1-year survival rate in the anti-MDA5 Abs group was lower than that in the anti-Jo-1 Abs group (<30% vs. 90%). Most of the mortalities in the anti-MDA5 Abs group occurred within the first 1–3 months after diagnosis. These findings are consistent with those of previous studies. Yoshida et al. [19] reported that patients with anti-MDA5 Abs have a higher 3-month mortality rate than those patients with anti-Jo-1 Abs. In the same study, the researchers reported that the patients with anti-MDA5 Abs presented with acute deterioration of ILD, which may occur within months from the onset of ILD, and exhibited poor responses to high-dose steroids with standard immunosuppressive therapies. Most of the patients died of RP-ILD and its complications, namely, secondary infections, pneumomediastinum, and acute respiratory distress syndrome. Several studies have reported that the short-term mortality rate of patients with RP-ILD with anti-MDA5 Abs is approximately 50%–70% and is especially high within the first 6–12 months after diagnosis [8, 20–22]. Compared with ILD in patients with anti-MDA5 Abs, ILD in patients with anti-Jo-1 Abs generally follows a more chronic course of disease [23]. Patients with anti-Jo-1 Abs often exhibit more positive responses to high doses of corticosteroids and other immunosuppressive drugs. Previous studies have also reported that patients with ILD with anti-Jo-1 Abs have relatively high rates of survival and favorable outcomes (>50% alive at the last follow-up (maximum: 18.5 years)) [24, 25], with 5-year mortality rates ranging from 30% to 50% [26, 27]. The distinct patterns of ILD and responses to therapy of patients with anti-MDA5 Abs and patients with anti-Jo-1 Abs may contribute to distinct prognoses and outcomes, as indicated by the findings of our study.

Previous studies have reported that some factors associated with poor ILD outcomes in patients with anti-MDA5 Abs include high serum ferritin (>500 ng/mL), high CRP (>1 mg/dL), and high KL-6 (>1000 U/mL) [28–30]. Lian et al. also reported that ferritin and LDH levels increased significantly in 66% and 54%, respectively, of patients with RP-ILD before their lung symptoms worsened [31]. Because RP-ILD is usually characterized by acute deterioration of pulmonary inflammation and rapid development of lung fibrosis, we considered that the aforementioned inflammatory biomarkers might reflect explosive inflammation in the lung parenchyma, which can lead to irreversible damage, resulting in respiratory failure, and eventually, mortality [32, 33].

Our study has some limitations. First, this was a single-country study conducted in Taiwan. However, this is the first retrospective study to compare the clinical characteristics and prognosis of patients with anti-Jo-1 and anti-MDA5 Abs. As the growing prevalence of patients with anti-MDA5 Abs in East Asia, this study may provide the demographics, symptom presentation, and prevalence of DM with different MSAs in Asian ethnicity. Second, unmeasured confounders may have produced bias in our results. Third, DM is an uncommon disease, the small sample size limited statistical analyses, we still identified significant differences in the clinical characteristics and 1-year mortality rates of patients with DM with anti-Jo-1 and anti-MDA5 antibodies. Additional multicenter studies with larger sample sizes are required to obtain more accurate information regarding the factors explored herein.

## 5. Conclusion

Compared with the patients with anti-Jo-1 Abs, the patients with anti-MDA5 Abs presented with more cutaneous ulcerative lesions and aggressive pulmonary inflammation and had a higher mortality rate, especially within the first 1–3 months after diagnosis. The results of our study may help clinicians recognize the clinical presentations of anti-MDA5 Ab–positive DM and diagnose RP-ILD earlier.

## Figures and Tables

**Table 1:** Univariate analysis comparing the clinical characteristics of the anti-Jo-1 Abs and anti-MDA-5 Abs groups.

	Anti-Jo-1 positive (*n* = 30)	Anti-MDA-5 positive (*n* = 8)	*P* value
Age at diagnosis (yrs)	63 ± 14	52 ± 16	0.068
Female, *n* (%)	21 (70%)	5 (62.5%)	0.685
Onset (<6mon)	24 (80%)	7 (87.5%)	0.627
ILD, *n* (%)	15 (65.2%)	7 (87.5%)	0.232
RP-ILD, *n* (%)	0 (0%)	6 (75%)	<0.001^∗^
HRCT pattern, *n* (%)			
None	15 (50%)	1 (12.5%)	0.036
NSIP	8 (26.7%)	1 (12.5%)	
UIP	1 (3.3%)	2 (25%)	
Ground glass	6 (20%)	4 (50%)	
Cardiac involvement	3 (13%)	0 (0%)	0.314
Fever	6 (26.1%)	6 (85.7%)	0.005^∗^
Muscle weakness	13 (56.5%)	6 (85.7%)	0.161
Heliotrope rash	2 (8.7%)	5 (71.4%)	0.001
Gottron's sign	11 (47.8%)	7 (100%)	0.014
Photosensitivity	0 (0%)	6 (85.7%)	<0.001^∗^
Cutaneous necrosis	0 (0%)	5 (71.4%)	<0.001^∗^
Mechanic hand	13 (56.5%)	6 (85.7%)	0.161
Periungual erythema	3 (13%)	5 (71.4%)	0.002^∗^
Auricular papules	0 (0%)	2 (28.6%)	0.008
Raynaud's phenomenon	5 (21.7%)	2 (28.6%)	0.708
Cutaneous vasculitis	0 (0%)	2 (28.6%)	0.008
Hoarseness	0 (0%)	3 (42.9%)	0.001^∗^
Dysphagia	1 (4.3%)	2 (28.6%)	0.061
Polyarthritis	21 (91.3%)	5 (71.4%)	0.176
Positive ANA	14 (63.6%)	2 (25%)	0.061
Maximal CK (IU/L)	2242 ± 3479	3389 ± 7568	0.579
LDH (U/L)	421 ± 177	766 ± 760	0.278
Ferritin (ng/mL)	823 ± 1196	1290 ± 202	0.570
AST (IU/L)	142 ± 156	406 ± 495	0.179
ALT (IU/L)	94 ± 93	219 ± 267	0.233
ESR (mm/hr)	38 ± 22	39 ± 23	0.930
CRP (mg/L)	1.8 ± 2.39	0.95 ± 0.62	0.362
PJP positive, *n* (%)	0 (0%)	3 (37.5%)	0.002
Pneumothorax, *n* (%)	0 (0%)	4 (50%)	<0.001^∗^
Hypertension	7 (23.3%)	1 (12.5%)	0.504
Diabetes mellitus	11 (36.7%)	8 (100%)	0.001^∗^
Respiratory failure, *n* (%)	3 (13%)	5 (62.5%)	0.006
Mortality, *n* (%)	2 (8.7%)	5 (62.5%)	0.002^∗^
Survival time (year)	0.93 ± 0.24	0.37 ± 0.44	0.014

Anti-MDA5: anti–melanoma differentiation-associated protein 5; ANA: antinuclear antibody; AST:aspartate aminotransferase; ALT: alanine transaminase; CK: creatine phosphokinase; CRP: C-reactive protein; ESR: erythrocyte sedimentation rate; HRCT: high-resolution computed tomography; ILD: interstitial lung disease; NSIP: nonspecific interstitial pneumonia; PJP: *Pneumocystis jirovecii* pneumonia; RP-ILD; rapid progressive interstitial lung disease; yrs: years; UIP: usual interstitial pneumonia. ^∗^*P* < 0.005.

## Data Availability

The underlying data supporting the results of our study were available on request. The corresponding authors (Tien and Cheng) are contacted to request the data.
